# CRISPR/Cas9 *ADCY7* Knockout Stimulates the Insulin Secretion Pathway Leading to Excessive Insulin Secretion

**DOI:** 10.3389/fendo.2021.657873

**Published:** 2021-06-11

**Authors:** Yazeid Alhaidan, Henrik Thybo Christesen, Elena Lundberg, Mohammed A. Al Balwi, Klaus Brusgaard

**Affiliations:** ^1^ Department of Clinical Genetics, Odense University Hospital, Odense, Denmark; ^2^ Department of Clinical Research, Faculty of Health Sciences, University of Southern Denmark, Odense, Denmark; ^3^ Department of Medical Genomics Research, King Abdullah International Medical Research Center, Riyadh, Saudi Arabia; ^4^ King Saud Bin Abdulaziz University for Health Sciences, Riyadh, Saudi Arabia; ^5^ Hans Christian Andersen Children’s Hospital, Odense University Hospital, Odense, Denmark; ^6^ Odense Pancreas Center, Odense, Denmark; ^7^ Institute of Clinical Science, Pediatrics, Umea University, Umeå, Sweden; ^8^ Department of Pathology and Laboratory Medicine, King Abdulaziz Medical City, NGHA, Riyadh, Saudi Arabia; ^9^ Near East University, Nicosia, Cyprus

**Keywords:** genetics, metabolomics, congenital hyperinsulinism, hyperinsulinemic hypoglycemia, pediatrics, adenylyl cyclase

## Abstract

**Aim:**

Despite the enormous efforts to understand Congenital hyperinsulinism (CHI), up to 50% of the patients are genetically unexplained. We aimed to functionally characterize a novel candidate gene in CHI.

**Patient:**

A 4-month-old boy presented severe hyperinsulinemic hypoglycemia. A routine CHI genetic panel was negative.

**Methods:**

A trio-based whole-exome sequencing (WES) was performed. Gene knockout in the RIN-m cell line was established by CRISPR/Cas9. Gene expression was performed using real-time PCR.

**Results:**

Hyperinsulinemic hypoglycemia with diffuse beta-cell involvement was demonstrated in the patient, who was diazoxide-responsive. By WES, compound heterozygous variants were identified in the adenylyl cyclase 7, *ADCY7* gene p.(Asp439Glu) and p.(Gly1045Arg). *ADCY7* is calcium-sensitive, expressed in beta-cells and converts ATP to cAMP. The variants located in the cytoplasmic domains C1 and C2 in a highly conserved and functional amino acid region. RIN-m^(-/-^ *^Adcy7^* ^)^ cells showed a significant increase in insulin secretion reaching 54% at low, and 49% at high glucose concentrations, compared to wild-type. In genetic expression analysis *Adcy7* loss of function led to a 34.1-fold to 362.8-fold increase in mRNA levels of the insulin regulator genes *Ins1* and *Ins2* (*p ≤* 0.0002), as well as increased glucose uptake and sensing indicated by higher mRNA levels of *Scl2a2* and *Gck via* upregulation of *Pdx1*, and *Foxa2* leading to the activation of the glucose stimulated-insulin secretion (GSIS) pathway.

**Conclusion:**

This study identified a novel candidate gene, *ADCY7*, to cause CHI *via* activation of the GSIS pathway.

## Introduction

Congenital hyperinsulinism (CHI) is a rare disease characterized with dysregulated insulin secretion from the pancreatic β-cells pancreatic leads to hyperinsulinemic hypoglycemia (1, 2). CHI is a heterogeneous disorder in terms of both phenotype and genotype. The rareness, severity, and complexity of CHI often lead to delayed and insufficient management with a high risk of subsequent brain damage mental retardation (3).

The estimated incidence of CHI is 1:40,000 in 50,000 newborns, but considerably higher in certain populations, e.g., 1:2,500 newborns in Saudi Arabia (4). The management of CHI is highly dependent on the major histological forms: focal or diffuse. ^18^F–fluoro-L-dihydroxyphenylalanine (18F–DOPA) PET/CT scan shows excellent performance in the identification of focal CHI, which is curable by limited pancreatic resections (5, 6). Milder forms of CHI response to medical treatment with diazoxide or somatostatin analogues, and an appropriate diet. Severe diffuse CHI with poor medical response, may require subtotal pancreatectomy, however imposing a high risk of subsequent diabetes (7). A medical approach is therefore encouraged, e.g. with long-acting octreotide (8).

Genetically, variants in at least nine genes have been identified as causes of CHI; ATP-Binding Cassette, subfamily C, member 8 (*ABCC8*) and Potassium Channel, Inwardly rectifying, subfamily J, member 11 (*KCNJ11*), which constitute the K^+^_ATP_ channel of the beta cell, accounts for most cases (9, 10). Less frequently, activating variants in the glucokinase gene (*GCK*) (11), or variants in the glutamate dehydrogenase gene (*GLUD1*) (12), or inhibitory variants in *HADH* (shortchain L-3-hydroxyacyl-coenzymeA dehydrogenase) (13), the monocarboxylase transporter (*SLC16A1*) (14), the uncoupling protein (*UCP2*) (15), *HNF4A* (hepatocyte nuclear factor 4 alpha) (16), and HNF1 homeobox alpha (*HNF1A*) (17). Recently, abnormalities in three new genes have been linked to CHI hexokinase 1 (HK1), phosphoglucomutase 1 (PGM1), and phosphomannomutase 2 (PMM2) genes (18–20). In addition, several syndromes are associated with CHI such as Beckwith–Wiedemann, Sotos, Kabuki, Usher, Timothy, Costello, Trisomy 13, and Mosaic Turner syndrome (21).

Despite the enormous efforts to understand this condition, up to 50% of patients still have negative investigations in the known pathogenic genes. This poses a challenge for both the geneticist and physician, as genotype-phenotype correlations provide a basis for targeted treatment. Here, we report a patient with CHI of unknown genetic cause by routine investigations. Functional studies carried out by generating a knockout cell line identified a novel candidate gene involved in hypersecretion of insulin.

## Materials and Methods

### Trio-Based Whole-Exome Sequencing

DNA was extracted from whole blood using The Maxwell^®^ RSC Blood DNA Kit from Promega. The proband was forwarded for a clinical CHI gene panel and subsequent a trio-based whole-exome sequencing (WES) approach. DNA samples obtained from our patient and his parents were subjected to exome capture using Roche NimbleGen SeqCap EZ Exome 3.5v Enrichment Kits (Roche, Hvidovre, Denmark) and sequencing was performed on the Illumina HiSeq 1500 platform. Raw reads were processed using the Burrows-Wheeler Alignment tool (BWA-MEM) v. 0.7.12, and the GATK Best Practice pipeline v. 3.3-0 was used for variant calling. A mean read depth of 63.6x was obtained.

### Data Analysis

VarSeqTM (Golden Helix, Inc., Bozeman, MT) was used for downstream filtering. All variants were first filtered with a minimum of 10× coverage, non-synonymous, and presented in the exome region or splice sites, which represented 94.4% of the targeted bases. Filtered variants were then processed twice, one for each parameter as previously described (22) The first parameter, which covers the possibility of a compound heterozygous, an autosomal recessive, a multifactorial, or *de novo*, was set to a population frequency of ≤ 0.01 (GenomAD and ExAC). The second parameter, which covers the dominant inheritance of single nucleotide polymorphisms (SNPs) and small insertions and deletions (INDELs), was set to a frequency of ≤ 0. 000025 for CHI.

To examine known causal genes that have been reported in the literature including related genes and pathways, a gene list was generated consisting of 6,264 genes categorized by disorders, pathways, expression, AmiGO terms, and other into 26 sublists (23). This was performed through an extensive literature review using PubMed, Ovid^®^, GeneCards^®^, and the National Center for Biotechnology Information (NCBI).Furthermore, we used gene and protein expression databases such as BioGPS and The Human Protein Atlas, protein interactions and gene network databases such as AmiGO, BioGRID, GIANT, KEGG, and Reactome, knockout mouse databases such as MGI and IMPC. However, filtering against the gene list will not replace the manual screening for all variants called; therefore, we did not consider the results of our gene list alone. Once the raw data were obtained, they were filtered and investigated individually. Variants went through serial steps ending up with a single nucleotide polymorphism variant as a potential explanation. Pathogenicity scores were determined by SIFT, PolyPhen-2, PANTHER, SNPs&GO, and nsSNPAnalyzer.

### Cell Culture

RIN-m cell line was purchased from American Type Culture Collection (LGC Standards GmbH, Wesel, Germany) and maintained in RPMI-1640 medium (ATCC) supplemented with 10% fetal bovine serum (FBS: Biological Industries) and 100 U penicillin and 100 µg/ml streptomycin (P4333:SIGMA) in a humidified incubator at 5% CO_2_ and 37°C.

### Knockout of *Adcy7* by CRISPR/Cas9

CRISPR/Cas9 guide-RNA targeting exon 5 in rat *Adcy7* (5′- TACCCATGGAGATGTGAGCT -3′) was manually designed using https://benchling.com and Alt-R^®^ S.p. Cas9 Nuclease 3NLS were ordered from (Integrated DNA Technologies, BVBA, Belgium). Lipofectamine^®^ RNAiMAX (Thermo Fischer, Naerum, Denmark) was used for transfection in Opti-MEM™ medium according to the manufacturer’s instructions. In short, knockout was performed by mixing 24 µl of 1 µM gRNA with 24 µl of 1 µM Cas9 Nuclease in 352 µl Opti-MEM^®^ Medium for 5 minutes to assemble the RNP complex. RNP complexes were mixed with 19.2 µl Lipofectamine^®^ RNAiMAX in 380 µl Opti-MEM^®^ Medium for 20 minutes to form transfection complexes. This was subsequently transferred to 1.6 ml of the complete medium without an antibiotic-antimycotic solution containing 6.4x10^5^ cells in 6 well plate. Cells were then incubated in a humidified incubator at 5% CO2 and 37°C for 72 hours prior to sequencing. Validated edited cells were then diluted and cultured at low cell concentration to isolate single cell colonies and establish a knockout *Adcy7* cell line, RIN-m^(-/^ *^-Adcy7^* ^)^.

### Sanger Sequencing

Gene editing was validated using BigDye Terminator v. 3.1 cycle sequencing kit and an ABI 3730xl capillary sequencer (Thermo Fischer, Naerum, Denmark). *Adcy7* primers targeting exon 5 in RIN-m cell line were ordered from Integrated DNA Technologies, BVBA, Belgium (forward 5′- ACAGGGAGGGCACATACTCT -3′, reverse 5′- AAATCCCCAGAGACACGCTC -3′).

### Stimulus Insulin Secretion of β-Cells

Both RIN-m^(-/^ *^-Adcy7^* ^)^ and RIN-m^WT^ were cultured for 24 hours prior to stimulation. This was performed by culturing 5x10^5^ cells in 2 ml of complete medium in 6 well plate. Krebs-Ringer-HEPES buffer (KRHB) was prepared (118 mM NaCl, 5.4 mM KCl, 2.4 mM CaCl_2_-2H_2_O, 1.2 mM MgSo_4_, 1.2 mM KH_2_Po_4_, 20 mM HEPES and 0.2% BSA) to be used for washing and stimulation experiments. Cells were washed twice with KRHB containing 1.1 mM glucose before starving for one hour in 1 ml KRHB containing 1.1 mM glucose. After starvation, cells were washed twice with KRHB containing 1.1 mM glucose and incubated in 2 ml KRHB containing 2mM or 10mM glucose to measure insulin secretion at low and high concentrations. Both cells and media were then collected to perform mRNA and insulin measurements. Each experiment was performed in triplicate.

### Real-Time PCR

Total RNA was extracted using an RNeasy Mini Kit (Qiagen, Copenhagen). The SuperScript^®^ III first-strand synthesis system (Thermo Fischer, Naerum, Denmark) was used for cDNA synthesis. The StepOne ™ system (Thermo Fischer, Naerum, Denmark) was used for real-time PCR using the TaqMan^®^ PreAmp Master Mix Kit and Expression Assay ID (*Adcy7*; Rn01538046_m1, *Actb*; Rn00667869_m1, *Ins1*; Rn02121433_g1, *Ins2*; Rn01774648_g1, *Prkaca*; Rn01432300_g1, *Prkacb*; Rn01748540_g1, *Rapgef4*; Rn01514839_m1, *Slc2a2*; Rn00563565_m1, *Pdx1*; Rn00755591_m1, *Gck*; Rn00561265_m1, and *Foxa2*; Rn01415600_m1) (Thermo Fischer, Naerum, Denmark). All data were analyzed using β-actin (*Actb*) expression as an endogenous control (**Supplemental Data**).

### Insulin Measurement by ELISA

A rat Insulin ELISA kit (cat n. ERINS) (Thermo Scientific, Naerum, Denmark) was used according to the manufacturer’s instructions. The Victor ™ X5 Multilabel Plate Reader was used to measure the absorbance (PerkinElmer, Skovlunde, Denmark).

### Data Analysis

All experiments were performed in triplicate. Changes in cycle threshold (CT) and total insulin concentrations between knockout and wild-type were calculated with an unpaired t-test. Data were presented as the mean and standard error of the means (mean ± SEM).

### Ethics

Oral and written consent was obtained from all participants. The study was approved by The Regional Ethical Committee of Southern Denmark (number. S-VF-20040235)

## Results

### Patient

A non-syndromic, Swedish Caucasian boy without Finnish inheritance was born at term, birth weight 3670** g**, birth length 52** cm**, with an uneventful neonatal period. He presented at age of 4 months weight 7460g (+1SDS), length 68** cm** (0 SDS) with hypoglycemic convulsions and loss of consciousness. At blood glucose 1.6mM, p-insulin was 19mU/L with no ketone body detected, confirming a diagnosis of CHI. An 18F–DOPA PET/CT scan showed diffuse pancreatic involvement.

Initially, he responded to treatment with diazoxide 10 mg/kg/day, divided into 3 doses. His growth followed +1 SDS in weight and 0 SDS in height according to the Swedish growth reference. His psychomotor development was normal. Diazoxide dose was reduced to 2 mg/kg/day from 4 to 7 years of age, but increased to 4mg/kg/day at the latest follow-up age 9 years.

No variants were found by a clinical next-generation sequencing panel of nine genes related to CHI as previously described (5).

### Sequencing Analysis

**Figure 1:**
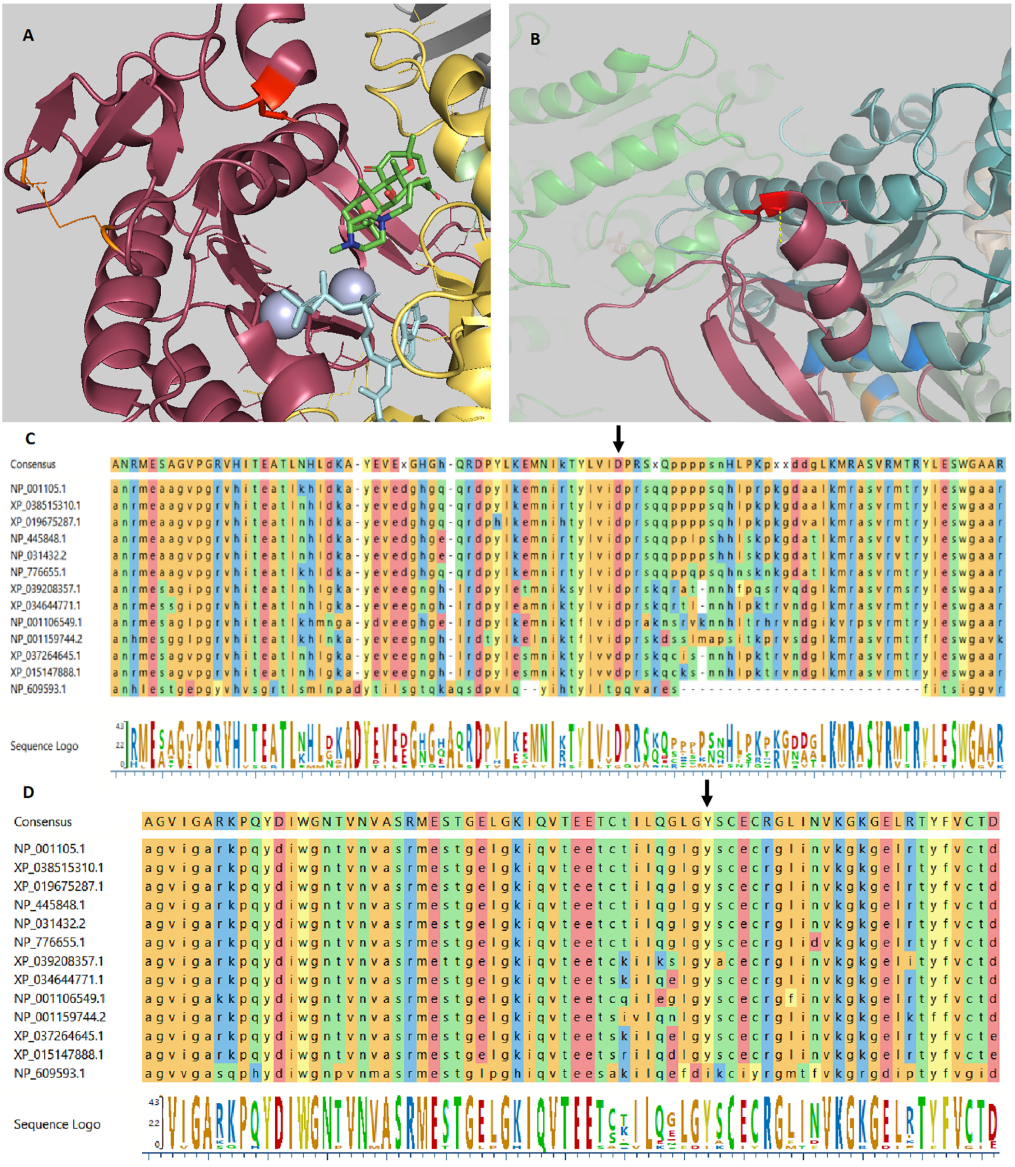
ADCY7 variants crystal structure. **(A)** The crystal structure of Gαs.VC1.IIC2 domain in complex with the substrate analog TNP-ATP based on pdb 2GVZ; ADCY5. The Asp439 residue is depicted as a red tooth stick. The adenylate cyclase inhibitor foskolin shown as green tooth sticks and the TNP-ATP substrate analog as aquamarine tooth sticks. The two manganese ions in grey. Two residues are depicted in orange are mutated in GUCY2D causing Leber congenital amaurosis 1. VC1 in salmon, IIC2 in yellow, Gαs in grey. Residues involved in VC1.IIC2 interaction shown as tooth sticks. **(B)** The crystal structure of the IIC2 domain based on pdb 6R4P; ADCY9. Gly1045 shown in red with a polar interaction indicated by a yellow dashed line to the backbone of residue Gly1043. Gly1043 allow a sharp turn to the loop connecting α-helix 15 and β-strand 19. Replacing Gly1045 with arginine can be predicted to prevent this turn. Residues depicted in blue are mutated in GUCY2D causing Leber congenital amaurosis 1. In the background the VC1 domain (green) can be seen. **(C)** Alignment of *ADCY7* sequences displaying conservation from homo sapiens at the top to Drosophila melanogaster at the bottom. Asp439 indicated by an arrow. **(D)** Alignment of *ADCY7* sequences displaying conservation from homo sapiens at the top to Drosophila melanogaster at the bottom displaying conservation of Gly1045 indicated by an arrow.

Trio WES analysis revealed compound heterozygous variants in the Adenylyl Cyclase 7 (*ADCY7*) gene. A paternal variant, NM_001114.4: c.1317C>A, p.(Asp439Glu) and a maternal variant, c.3133G>A, p.(Gly1045Arg), were found. These variants are located in the cytoplasmic domains C_1_ and C_2_, respectively, (**Figure 1**) in a highly conserved amino acid region (24, 25). The ConSurf server for the identification of functional regions in proteins predicted both amino acids to play a functional role and being exposed to an interaction site. The prediction software SIFT, PolyPhen-2, PANTHER, SNPs&GO, and nsSNPAnalyzer predicted both variants to be deleterious. *ADCY7* is reported to be expressed in pancreatic tissue with other isoforms *ADCY1*, *ADCY3*, *ADCY5*, and *ADCY9* (26). Adenylyl cyclases (ACs) are a family of ten different mammalian isoforms that converts ATP to cyclic AMP. *ADCY7* is a membrane-bound member of this family, reported to be inhibitable by calcium.

### Protein Sequence Analysis

The three-dimensional protein structure of candidate genes was analyzed using PyMOL v.1.7.4 (Schrödinger, New York, USA). (PDB-ID: 2GVZ).

### Cell Line Establishment

**Figure 2:**
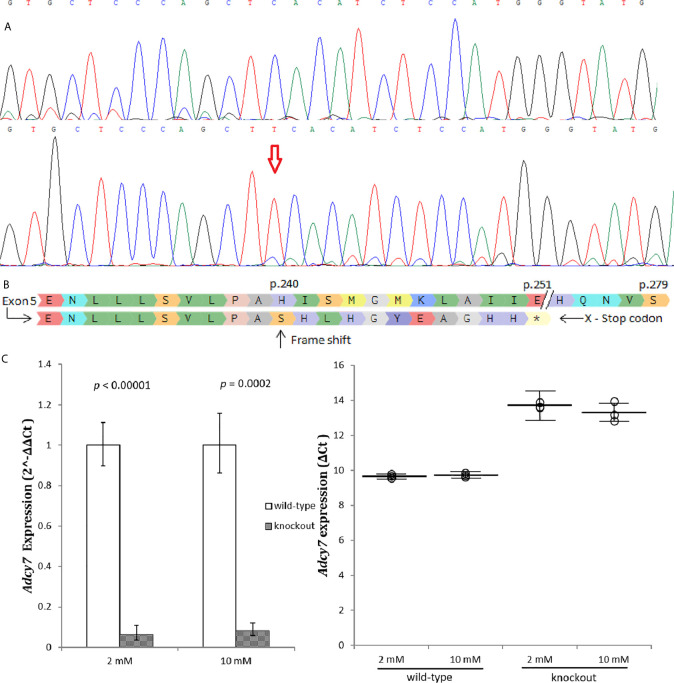
*Adcy7* knockout establishment. **(A)** Sanger sequencing wild-type RIN-m^(^ *^Adcy7^* ^/^ *^Adcy7^* ^)^ (Top) compared to the mutant cell line (bottom) confirmed an insertion creating a frameshift. **(B)** Amino acid sequencing with a reference (top) shows indel creating a frameshift causing a premature termination codon located in 11 codons downstream. **(C)** *Adcy7* mRNA levels established by quantitative RT-PCR indicating a very low expression level. The CT values were normalized to β-actin to yield ΔCT and 2^-ΔΔCT values presented in dots and bar chart, respectively. Each dot represents an individual sample, whereas the horizontal lines represent the mean and standard error of the mean. ΔCT values were then calculated and presented as 2^-ΔΔCT values (fold change) in a bar chart. Unpaired t-test with delta CT and SE ± values were used for statistic test.

An *Adcy7* knockout cell line, RIN-m^(-/^ *^-Adcy7^* ^)^, was established and validated, (**Figure 2A**). A one base insertion located in exon 5 created a frameshift leading to generation of a premature termination codon located 11 codons downstream (**Figure 2B**). In addition, the *Adcy7* mRNA level determined by real-time PCR confirmed the loss of the expression as a result of degradation *via* nonsense-mediated mRNA decay (**Figure 2C**).

### *Adcy7* Knockout Shows an Excessive Insulin Expression and Secretion

**Figure 3:**
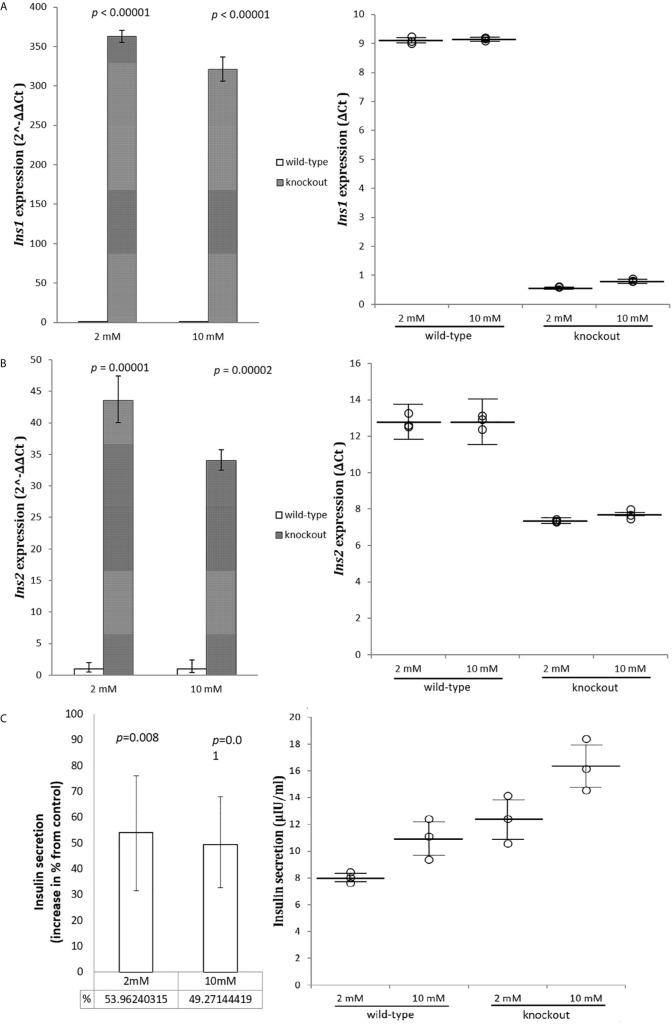
Insulin genes and secretion. RIN-m(^-/-^ *^Adcy7^*) mRNA level established by quantitative RT-PCR. The CT values were normalized to β-actin to yield ΔCT and 2^-ΔΔCT values presented in dots and bar chart, respectively. Each dot represents an individual sample, whereas the horizontal lines represent the mean and standard error of the mean. ΔCT values were then calculated and presented as 2^-ΔΔCT values (fold change) in a bar chart. **(A)** presents the mRNA level of *Ins1* when incubated in low glucose level 2mM and high glucose level 10mM, while **(B)** shows the expression level of *Ins2* under the same condition. **(C)** illustrates insulin secretion when incubated for one hour with 2mM glucose and 10mM glucose compared to the control. Unpaired t-test with mean of ELISA or delta CT and SE ± values were used for statistic test.

To investigate whether *Adcy7* influences the insulin secretion genes *Ins1* and *Ins2*, cells were incubated in low 2 mM and high 10 mM glucose concentration and mRNA was measured. The levels of both *Ins1* and *Ins2* mRNA displayed a statistically significant increase in absence of *Adcy7* gene expression. The regulator of insulin gene expression, *Ins1* was significantly increased by 362.8 fold (*p*<0.00001) at low glucose media 2 mM, and by 320.9 fold (*p*<0.00001) at high glucose media 10 mM (**Figure 3A**). The secretion regulator *Ins2* was also dramatically increased by 43.6 fold (*p*=0.00001) at low glucose concentration and by 34.1 fold (*p*=0.00002) at high glucose concentration for RIN-m^(-/^ *^-Adcy7^* ^)^ cells compared to the wild-type (**Figure 3B**).

Modified cells showed a significant increase in insulin secretion reaching 54% at low, and 49% at high glucose concentration, respectively, compared to the wild-type (*p*=0.008, *p*=0.01), (**Figure 3C**).

### Insulin Secretion Pathway

As *Adcy7* converts ATP to cyclic AMP and pyrophosphate, we examined whether the loss of the *Adcy7* function influences insulin secretion *via* the cAMP pathway.

**Figure 4:**
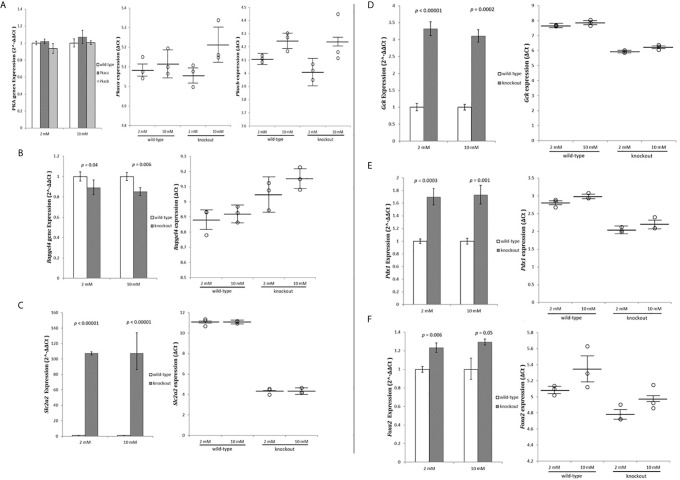
Exome data analysis strategy. RIN-m^(-/-^ *^Adcy7^* ^)^ mRNA level under 2mM glucose and 10mM glucose concentration established by quantitative RT-PCR. The CT values were normalized to β-actin to yield ΔCT and 2^-ΔΔCT values presented in dots and bar chart, respectively. Each dot represents an individual sample, whereas the horizontal lines represent the mean and standard error of the mean. ΔCT values were then calculated and presented as 2^-ΔΔCT values (fold change) in a bar chart. **(A)** PRKACs alpha (*Prkaca*) and beta (*Prkacb*) expressions, **(B)** *Rapgef4*, **(C)** *Slc2a2*, **(D)** *Gck*, **(E)** *Pdx1*, **(F)** *Foxa2*. Unpaired t-test with delta CT and SE ± values were used for statistic test.

Protein Kinase cAMP-Activated Catalytic Subunits alpha (*Prkaca*) and beta (*Prkacb*) and Rap Guanine Nucleotide Exchange Factor 4 (*Rapgef4*) mRNA, which are highly expressed in pancreatic tissue, were measured as per their role on cAMP activation (27). mRNA levels for the PRKACs subunits indicated no change in the expression level (**Figure 4A**) while *Rapgef4* decreased slightly, but significant (**Figure 4B**). These results indicated no activation of the cAMP pathway in RIN-m^(-/^ *^-Adcy7^* ^)^ cells as the cause to increase insulin secretion.

Next, we investigated whether the glucose stimulated-insulin secretion (GSIS) pathway was activated. The glucose uptake and the sensing genes Solute Carrier Family 2 Member 2 (*SCL2A2*) and Glucokinase (*GCK*) mRNA levels were measured. Glucose transporter member 2 (*Glut2*) protein, was extremely upregulated (107.3 fold - *p*<0.00001) and (107.5 fold - *p*<0.00001) for both low and high glucose concentrations, respectively (**Figure 4C**). Furthermore, glucokinase protein, which phosphorylates glucose in the first step of GSIS in β-cells, was also significantly upregulated (3.32 fold - *p*<0.00001) during low glucose conditions and (3.1 fold - *p*=0.0002) during high glucose level (**Figure 4D**).

To investigate the causes of the extreme upregulation of insulin and GSIS genes, Pancreatic and duodenal homeobox 1 (*Pdx1*), and Forkhead Box Protein A2 (*Foxa2*) genes expression were measured. *Pdx1* was significantly up-regulated by 1.7 fold (*p*=0.0003) in 2 mM glucose and 1.73 fold (*p*=0.001) in 10 mM compared to the wild-type (**Figure 4E**). On the other hand, *Foxa2*, which binds to *Pdx1* to regulate genes responsible for maintaining the mature β-cell function, were slightly but significantly increased by 1.23 fold (*p*=0.006) and 1.29 fold (*p*=0.05) when incubated with 2 mM and 10 mM glucose concentration, respectively, compared to the control (**Figure 4F**).

## Discussion

In this article, we identified *ADCY7* as a novel candidate genetic cause of CHI. *ADCY7* encodes a membrane-bound adenylate cyclase that converts ATP to cyclic AMP and pyrophosphate. We showed that *Adcy7* loss of function leads to excessive insulin secretion and activated the glucose stimulated-insulin secretion pathway.

Mammalian adenylyl cyclases (ACs) consist of nine membrane-bound proteins AC isoforms, AC1–AC9, coded by *ADCY1*-*ADCY9* (28). ACs are constructed by two clusters of six transmembrane domains each followed by a cytoplasmic domain C_1_ and C_2_ (29). Our patient’s *ADCY7* variants were predicted to disrupt the function of the highly conserved domain C_1_ and C_2_ justifying further loss-of-function studies. The Asp439 variant is shown to be reside in the gate to the activating domain in VC1 thus likely influencing regulation of substrate uptake (**Figure 1A**). Gly1045 allow a sharp turn of the loop connecting α-helix 15 and β-strand 19 necessary for IIC2 confirmation (**Figure 1B**).

Several studies have been linking AC members with insulin secretion and sensitivity. For instance, A gain of function of *Adcy3* in mutagenized mice resistant to diet-induced obesity caused a reduction in both body weight, fat mass, insulin, and glucose levels compared to wild-type when subjected to high fat diet (30). On the contrary, whole-body *Adcy5* knockout mice exhibited lower weight, lower fasting glucose, improved glucose tolerance, and increased insulin sensitivity (31). In pancreatic islet study by Hodson and his colleagues, *ADCY5* silencing led to impaired glucose-induced cAMP increases and lower ATP concentrations at high glucose levels, in keeping with reduced mRNA expression of *ADCY5* in patients with the *ADCY5* SNP rs11708067, which increases the risk of type 2 diabetes (32). At least in part, differences in tissue and cell type-specific localization of the individual AC may explain the diversity of regulatory features by different AC members in cAMP pathway signaling (24, 33). Currently, no study reported the association of *ADCY7* with insulin secretion and sensitivity. In the present study, *ADCY7* loss of function was associated with increased insulin secretion, but not with evidence of cAMP alterations at glucose concentrations up to 10 mM. This was in line with findings of Hodson et al., where cAMP changes after *ADCY5* silencing only occurred at higher glucose levels (32). More studies are needed to understand the differences between the different ACs in glucose metabolism. Compared to the wild-type, our RIN-m^(-/^ *^-Adcy7^* ^)^ demonstrated an increase in the acute insulin activity leading to a 54% higher secretion during hypoglycemia *in vitro*. These results mimic the pathophysiology of CHI.

Genetic analysis using RT-PCR gene expression indicated that *Adcy7* loss of expression upregulates the insulin regulator genes, *Ins1* and *Ins2*, as well as glucose uptake and sensing by regulating *Scl2a2* and *Gck via* upregulation of *Pdx1*.

*PDX1* is a transcriptional factor that regulates the insulin gene. Transfection of a small interfering RNA specific for *Pdx1* in pancreatic islets insulinoma cell line shows that *Pdx1* directly regulates the insulin transcription promotor (34). Dox-induced *Pdx1* expression in embryonic stem cells increased *Ins1* and *Ins2* mRNA compared to the non-induced cells and corresponding upregulated by 140% compared to the insulinoma cell line βTC6 (35).

Insulin secretion from the pancreatic β cell is a tightly regulated process by insulin-tropic factors stimulating intracellular cAMP, or by glucose sensing and uptake. We examined whether intracellular cAMP activation is responsible for increasing insulin secretion in RIN-m^(-/^ *^-Adcy7^* ^)^ cells, similar to the role of *ADCY3* haploinsufficiency in a previous report (36). The mRNA level for PRKACs subunits did not show significant changes, while *Rapgef4* decreased only slightly. These results indicated no robust evidence of a cAMP activation pathway in RIN-m^(-/^ *^-Adcy7^* ^)^ cells up to 10 mM glucose concentration. Subsequently, the expression analysis of key components of glucose uptake and sensing, *Scl2a2*, and *Gck*, revealed an extreme increase in mRNA levels leading to the activation of the glucose stimulated-insulin secretion (GSIS) pathway. This due to overexpression of *PDX1* gene (37), seen in RIN-m^(-/^ *^-Adcy7^* ^)^. In *Pdx1* overexpressed mouse *βPdx1; Ins2^Akita^*, insulin secretion, *Gck* mRNA, and *Slc2a2* localization was significantly increaseed compared to the non-modified *Ins2^Akita^* mouse (38). *Foxa2* on the other hand, which binds to *Pdx1* to regulate genes responsible for maintaining mature β-cell function (39), was also significantly increased. However, the exact mechanism by which *ADCY7* leads to excessive insulin secretion through GSIS is still not explained by only increasing *SLC2A2* and *GCK* expression during hypoglycemia conditions. In addition, the similarity of insulin secretion percentage between two glucose concentrations compared to wild-type as well as the expression changes seen in this study may also suggest loss of glucose sensitivity rather than an increased glucose phosphorylation. Further analyses with e.g. KCl, sulphonylurea and diazoxide, Gck activity, downstream analyses of both GSIS and cAMP pathways using a full gene expression profile as well as a protein study are needed to fully explain the *ADCY7* mechanism.

RIN-m rat pancreas/islet cell line is a well-characterized cell line that secretes insulin with some concerns raised regarding GSIS and glucose sensitivity. Due to the limitation of a stable and well-characterized human pancreatic cell line, several published studies used RIN-m to investigate target genes concerning insulin secretion and GSIS (40–42). Santina Bruzzone et al. presented the effects of Abscisic acid (ABA) on insulin secretion using RIN-m and human pancreatic islets, concluding that they both show the same effect mediating cAMP on different glucose level (43). In addition, pre-treatment and starvation have shown different insulin reactions toward glucose in the RIN-m cell line (42). In this study, RIN-m wild-type and RIN-m^(-/^ *^-Adcy7^* ^)^ cultured in 10 mM demonstrated 36.3%, and 32.3% higher in insulin secretion compared to 2 mM, respectively. Nonetheless, the *ADCY7* role in glucose induced insulin secretion needs to be further tested in other cell types, e.g. human pancreatic islets, EndoC-βH1 and rat pancreatic cells, BRIN-BD11 and INS-1.

In conclusion, our study identified a novel candidate gene *ADCY7* to cause CHI. The loss of *Adcy7* function in the RIN-m cell line resulted in higher insulin secretion. The complexity of the relationship between *ADCY7* and insulin secretion was at least in part explained by the changes in glucose sensing and glucose uptake in β-cells, which regulates insulin secretion *via* the glucose stimulated-insulin secretion pathway. Our finding will potentially open new doors for future work on the adenylyl cyclase complex with an insulin-signaling pathway to obtain better understanding of signaling pathway; secondly to develop a new therapeutic treatment triggering this pathway for patients with diabetes or congenital hyperinsulinism.

## Data Availability Statement

The original contributions presented in the study are included in the **Supplementary Material**, further inquiries can be directed to the corresponding author.

## Ethics Statement

The study was approved by The Regional Ethical Committee of Southern Denmark (number. S-VF-20040235). Written informed consent to participate in this study was provided by the participants’ legal guardian/next of kin.

## Author Contributions

KB, HC, and MB were involved in planning and supervising the project. HC and EL collected and provided the clinical data. YA and KB designed the experiments. YA performed the experiments and analyzed the data. YA drafted the manuscript. All authors contributed to the article and approved the submitted version.

## Funding

This project was funded by Region of Southern Denmark and The University of Southern Denmark.

## Conflict of Interest

The authors declare that the research was conducted in the absence of any commercial or financial relationships that could be construed as a potential conflict of interest.
